# Genetic associations in the gut–lung axis: a Mendelian randomization study and *in vitro* assessment of sodium butyrate in non-small cell lung cancer

**DOI:** 10.3389/fgene.2026.1900040

**Published:** 2026-10-16

**Authors:** Weili Yi, Li Qin, Dongning Huang, Fang Wu

**Affiliations:** 1 Department of Radiation Oncology, The First Affiliated Hospital of Guangxi Medical University, Nanning, China; 2 Department of Oncology, The Fourth Affiliated Hospital of Guangxi Medical University, Liuzhou, China

**Keywords:** FinnGen, gut microbiota, macrophages, Mendelian randomization, non-small cell lung cancer, sodium butyrate

## Abstract

**Background:**

The gut microbiota may influence host immunity, but the relationship between specific microbial genera and non-small cell lung cancer (NSCLC) risk remains uncertain. We evaluated genetically proxied microbial abundance and conducted separate exploratory experiments with sodium butyrate (NaB).

**Methods:**

Two-sample Mendelian randomization (MR) used genus-level MiBioGen summary statistics and FinnGen/OpenGWAS NSCLC data (finn-b-C3_LUNG_NONSMALL; 1,627 cases and 217,165 controls). Instruments were selected at P < 1 × 10^−5^ with linkage disequilibrium clumping (r^2^ < 0.001; 10,000 kb) and F > 10. Inverse variance weighting (IVW) was the primary estimator; Benjamini–Hochberg false discovery rate (BH-FDR) correction was applied across 119 genera. Sensitivity analyses included MR-Egger, weighted median, mode-based methods, heterogeneity, pleiotropy and leave-one-out analyses. NaB, used as a representative microbial metabolite rather than a taxon-specific product, was evaluated at 1 mM for 24 h in tumor-conditioned medium (TCM) experiments.

**Results:**

Six genera showed nominal inverse IVW associations with NSCLC risk (P = 0.0120–0.0498): Alistipes, Methanobrevibacter, *Bacteroides*, Ruminococcus gauvreauii group, Ruminococcus 1 and *Peptococcus*. None survived BH-FDR correction (q = 0.6072–0.9819). Sensitivity tests did not detect heterogeneity or directional pleiotropy for these genera, but alternative estimators and leave-one-out analyses did not consistently retain nominal significance. In THP-1-derived macrophages, NaB treatment altered polarization-associated transcripts, reduced the TCM-associated CCK-8 signal and increased acetylated histone H3 (Ac-H3) relative to TCM alone.

**Conclusion:**

The MR analysis identifies six suggestive microbial associations with NSCLC risk that require independent replication. Complementary NaB experiments show changes in macrophage-associated transcripts, metabolic viability and histone acetylation, providing a basis for further study of microbial metabolites in the tumor microenvironment.

## Introduction

Lung cancer remains a leading cause of cancer mortality (Sung et al., 2021; Bade and Dela Cruz, 2020). Non-small cell lung cancer (NSCLC), comprising mainly adenocarcinoma and squamous cell carcinoma, accounts for most lung cancers (Duma et al., 2019; Herbst et al., 2018). Treatment depends on disease stage and molecular features, with surgery, systemic therapies and immune checkpoint inhibitors contributing to care (Reck and Rabe, 2017; Hirsch et al., 2017). Resistance and immune escape remain important challenges (Sharma et al., 2017; O’Donnell et al., 2019). These observations motivate investigation of host and environmental factors that may influence lung cancer biology.

The gastrointestinal tract contains a diverse microbial community (Sender et al., 2016). Microbial metabolites and immune signals can influence host physiology beyond the intestine (Fan and Pedersen, 2021; Cho and Blaser, 2012). The gut–lung axis provides a framework for investigating these distant interactions and their relevance to pulmonary disease (Dang and Marsland, 2019).

Tumor immune escape involves interactions between malignant cells and the surrounding immune compartment (Sharma et al., 2017; O’Donnell et al., 2019). Clinical studies have linked gut microbiome composition with response to PD-1-based immunotherapy, including studies in NSCLC (Routy et al., 2018; Jin et al., 2019). Antibiotic exposure has also been associated with poorer outcomes during immune checkpoint inhibitor treatment (Pinato et al., 2019; Derosa et al., 2018). These observational findings concern treatment response or survival, and may be influenced by treatment indication and other confounders. The relationship with NSCLC susceptibility remains a separate question.

Observational studies of the microbiome and NSCLC are vulnerable to confounding by diet, smoking, antibiotic exposure and environmental factors. Reverse causation is also possible because cancer and its systemic effects may alter the gut microbiome.

Mendelian randomization (MR) uses genetic variants as instrumental variables for an exposure (Richmond and Davey Smith, 2022; Sanderson et al., 2022). Under assumptions of instrument relevance, independence from confounders and absence of effects on the outcome through other pathways, MR can inform causal hypotheses. This approach complements observational microbiome studies by using genetic proxies for microbial abundance.

We used two-sample MR to examine associations between genetically proxied gut microbial abundance at genus level and NSCLC risk (Figure 1). Separately, we conducted exploratory experiments using sodium butyrate (NaB), a representative microbial metabolite, to examine polarization-associated transcripts, metabolic viability and histone acetylation in THP-1-derived macrophages exposed to A549-derived TCM. The genetic and cellular analyses address microbial associations and metabolite responses, respectively.

**FIGURE 1:**
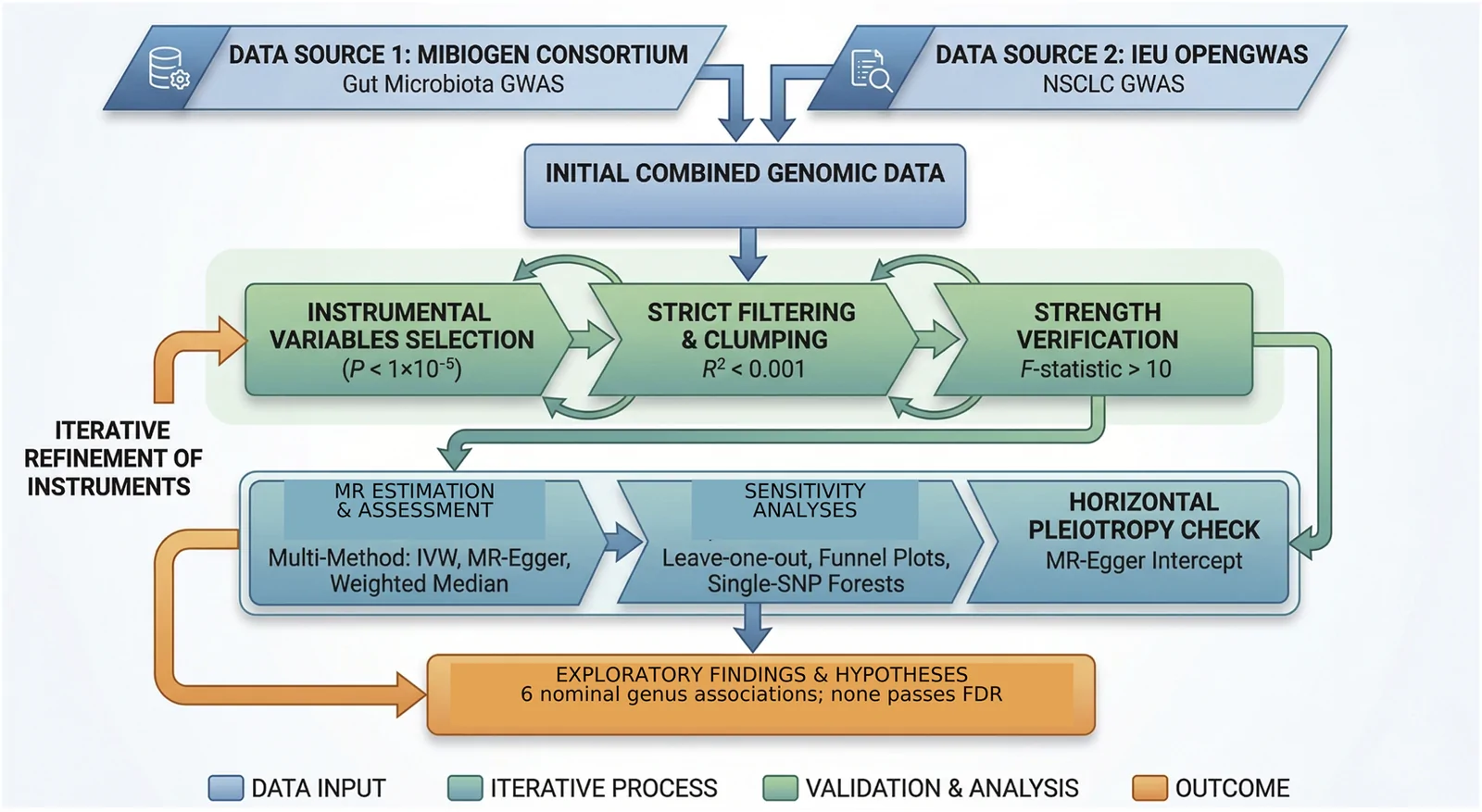
Study design and analysis workflow. MiBioGen exposure data comprise 18,340 participants from 24 cohorts. The outcome is FinnGen/OpenGWAS C3_LUNG_NONSMALL. Primary IVW tests cover 119 genera and are corrected using BH-FDR. NaB cell experiments constitute a separate exploratory arm.

## Materials and methods

### Study design and MR assumptions

The two-sample MR framework was based on three assumptions: genetic instruments are associated with microbial abundance; they are independent of confounders of the exposure–outcome relationship; and they affect NSCLC only through the exposure (Richmond and Davey Smith, 2022; Sanderson et al., 2022; Skrivankova et al., 2021). Figure 1 summarizes the workflow. Reporting followed STROBE-MR (Skrivankova et al., 2021). The genetic analysis and NaB experiments address related but distinct questions and were interpreted separately.

### Microbiome GWAS data

Exposure summary statistics were obtained from the MiBioGen consortium meta-analysis of 16S rRNA gene profiles and genotypes in 18,340 participants from 24 cohorts (Kurilshikov et al., 2021). MiBioGen included multiple ancestry groups, predominantly European. This analysis used genus-level traits and included 119 genera with retained instruments and available IVW results.

## NSCLC outcome data

Outcome summary statistics were obtained from FinnGen release 5 (DF5) through OpenGWAS (finn-b-C3_LUNG_NONSMALL), with 1,627 cases and 217,165 controls (Kurki et al., 2023; IEU OpenGWAS, 2026; Release 5: release history, endpoint and control definitions, and summary-statistics manifest, 2026). The DF5 definition uses the Finnish ICD-10 matching expression C34.[123457] in both the hospital discharge and causes-of-death fields (Release 5: release history, endpoint and control definitions, and summary-statistics manifest, 2026). Cancer-registry topography and morphology fields are blank for this endpoint. The control definition specifies no additional cancer exclusion; controls were therefore not necessarily free of other cancers. The summary statistics use GRCh37 coordinates. The analysis used the overall NSCLC endpoint without histology-specific stratification. The official historical definition files, R5 manifest and further provenance details are provided in the supplement.

### Instrument selection and harmonization

Because few microbiome variants reach conventional genome-wide significance, an exposure association threshold of P < 1 × 10^−5^ was used to increase instrument availability (Burgess and Thompson, 2011; Pierce et al., 2011). LD clumping used a European reference panel with r^2^ < 0.001 and a 10,000 kb window. A European panel was selected because FinnGen is of Finnish European ancestry and European participants predominate in MiBioGen. Alleles were harmonized between the exposure and outcome data using TwoSampleMR action 2. The retained dataset contained 1,421 SNP–exposure records across 119 genera. Retained instruments, harmonization outputs and the original analysis scripts are provided in the Supplementary Material.

### MR estimation and statistical analysis

MR analyses were conducted in R 4.4.2 using TwoSampleMR. Instruments with F < 10 were excluded. The original per-SNP F statistics and the approximation F = (βexposure/SEexposure)^2^ are reported in Supplementary Table S3. IVW was the primary estimator, with MR-Egger, weighted median, simple mode and weighted mode as complementary methods. BH-FDR correction was applied to all 119 primary IVW P values; Bonferroni-adjusted P values are also provided (Benjamini and Hochberg, 1995). Associations with unadjusted P < 0.05 but q ≥ 0.05 are described as nominal or suggestive. Cochran’s Q, the MR-Egger intercept and MR-PRESSO global test evaluated heterogeneity and pleiotropy. Leave-one-out analyses used multiplicative random-effects IVW, with the residual variance factor bounded below by 1. Reverse MR and Steiger directionality testing were not performed. Original MR-PRESSO outputs and analysis scripts are included in the Supplementary Material.

### Cell culture and tumor-conditioned medium

The human NSCLC cell line A549 and human monocyte cell line THP-1 were obtained from the Cell Bank of the Chinese Academy of Sciences (Shanghai, China). Cells were cultured in RPMI-1640 medium (Servicebio, Wuhan, China) supplemented with 10% fetal bovine serum (Excell Bio, Taicang, China) and 1% penicillin–streptomycin at 37 °C and 5% CO_2_. For TCM preparation, A549 cells were grown to approximately 80% confluence, transferred to fresh complete medium and cultured for 48 h. Supernatants were collected, centrifuged to remove debris, filtered through a 0.22 μm membrane and stored at −80 °C. THP-1 cells were treated with 100 ng/mL phorbol 12-myristate 13-acetate (PMA; Solarbio, Beijing, China) for 24 h, followed by PMA removal and 24 h of rest in fresh medium to obtain M0 macrophages. The experimental groups were Control, 50% TCM and 50% TCM plus 1 mM NaB (Macklin, Shanghai, China). TCM and NaB were applied simultaneously for 24 h.

#### Molecular and viability assays

Total RNA was extracted using TRIzol (Vazyme, Nanjing, China). Reverse transcription and RT-qPCR used HiScript III RT SuperMix and ChamQ Universal SYBR qPCR Master Mix (Vazyme), with primers synthesized by Sangon Biotech (Shanghai, China). RT-qPCR measured TNF-α, CD86, iNOS, Arg-1, TGF-β and CD206 transcripts in THP-1-derived macrophages. Relative expression was calculated by the 2^−ΔΔCT^ method using GAPDH as the reference gene. For each biological sample, ΔCt was calculated as target-gene Ct minus the matched GAPDH Ct. The mean ΔCt of the Control group was used as the calibrator to obtain ΔΔCt and 2^−ΔΔCT^. Ct values and the corresponding calculations are provided in the experimental source tables. Target-primer sequences are listed in Supplementary Table S11.

CCK-8 (Biosharp, Beijing, China) was performed on THP-1-derived macrophages after Control, 50% TCM or 50% TCM plus 1 mM NaB treatment for 24 h. OD450 values were background-corrected using the mean of three blank wells and expressed as a percentage of the control mean. CCK-8 was used as a measure of metabolic viability.

For Western blotting, proteins from THP-1-derived macrophages were extracted with RIPA buffer (Beyotime, Shanghai, China). Ac-H3 (approximately 17 kDa; 1:1,000; ABclonal, Wuhan, China) was detected and normalized to GAPDH (approximately 37 kDa; 1:5,000; Proteintech, Wuhan, China) using ECL (Vazyme). ImageJ was used for densitometry. Ac-H3/GAPDH ratios were normalized to the control mean.

### Statistical analysis of cell experiments

The biological replicate was the unit of analysis: n = 4 per group for RT-qPCR, n = 6 for CCK-8 and n = 3 for Western blotting. Technical wells were not counted as additional biological replicates. Data are presented as mean ± SD. For each of the eight experimental endpoints, the three groups were compared using ordinary one-way ANOVA followed by Tukey’s test for all three pairwise comparisons. Analyses used unrounded values. qPCR tests used relative expression (2^−ΔΔCT^) calculated with sample-matched GAPDH normalization. CCK-8 tests used background-corrected values normalized to the control mean, and blot tests used normalized Ac-H3/GAPDH ratios. Tukey adjustment was applied within each endpoint; no additional adjustment across transcripts or experimental endpoints was applied. Supplementary Tables S12–S14 report individual values, means, SDs, ANOVA statistics, degrees of freedom and Tukey-adjusted P values.

## Results

### Instrument characteristics

The retained harmonized dataset contained 1,421 SNP–exposure records representing 1,416 distinct rs identifiers across 119 genera (Supplementary Table S3). For the six nominally associated genera, instrument counts ranged from 8 to 16, and individual F statistics ranged from 18.04 to 32.25 (Tables 1 and 2). All retained instruments exceeded the conventional F = 10 threshold.

**TABLE 1:** Nominal IVW associations and correction across 119 genera.

Genus	SNPs	OR (95% CI)	Raw P	FDR q
Alistipes	12	0.574 (0.352–0.934)	0.025481	0.6072
Methanobrevibacter	8	0.751 (0.589–0.958)	0.020933	0.6072
*Bacteroides*	10	0.629 (0.396–1.000)	0.049819	0.9819
Ruminococcus gauvreauii group	12	0.671 (0.478–0.942)	0.021330	0.6072
Ruminococcus 1	13	0.680 (0.485–0.954)	0.025513	0.6072
*Peptococcus*	16	0.790 (0.657–0.950)	0.012047	0.6072

IVW, inverse variance weighted; SNP, single nucleotide polymorphism; OR, odds ratio; CI, confidence interval; FDR, false discovery rate. Raw P values are unadjusted; q values use the Benjamini–Hochberg procedure across 119 genera. All six Bonferroni-adjusted P values equal 1.000. The odds ratios correspond to the exposure scale of the source GWAS.

**TABLE 2:** Instrument strength and sensitivity tests.

Genus	Individual F range	IVW Q P	Egger intercept P	PRESSO global P
Alistipes	18.04–23.37	0.2469	0.7643	0.249
Methanobrevibacter	19.36–24.05	0.5130	0.8525	0.506
*Bacteroides*	20.04–26.18	0.3190	0.5647	0.364
Ruminococcus gauvreauii group	18.86–25.73	0.4965	0.7426	0.497
Ruminococcus 1	19.03–23.56	0.5804	0.3582	0.586
*Peptococcus*	19.50–32.25	0.8964	0.8993	0.901

IVW, inverse variance weighted; Q, Cochran’s heterogeneity statistic; MR-PRESSO, Mendelian Randomization Pleiotropy RESidual Sum and Outlier. F ranges summarize individual instruments for each genus. The table reports P values for the IVW Q test, MR-Egger intercept test and MR-PRESSO global test; MR-PRESSO values retain the precision of the original resampling output. Full statistics, degrees of freedom, intercepts and standard errors are provided in Supplementary Table S5.

### Nominal associations and multiple testing

Six genera showed inverse IVW associations at unadjusted P < 0.05: Alistipes (OR 0.574, 95% CI 0.352–0.934), Methanobrevibacter (0.751, 0.589–0.958), *Bacteroides* (0.629, 0.396–1.000), Ruminococcus gauvreauii group (0.671, 0.478–0.942), Ruminococcus 1 (0.680, 0.485–0.954) and *Peptococcus* (0.790, 0.657–0.950) (Table 1; Figure 2). For *Bacteroides*, the unrounded upper CI was 0.999643 and P was 0.049819. None survived BH-FDR correction across 119 genera: q = 0.6072 for five genera and q = 0.9819 for *Bacteroides*. None survived Bonferroni correction. No positive IVW association reached nominal P < 0.05 in this dataset; this does not establish that risk-increasing taxa do not exist. All 119 primary results are provided in Supplementary Table S1.

**FIGURE 2:**
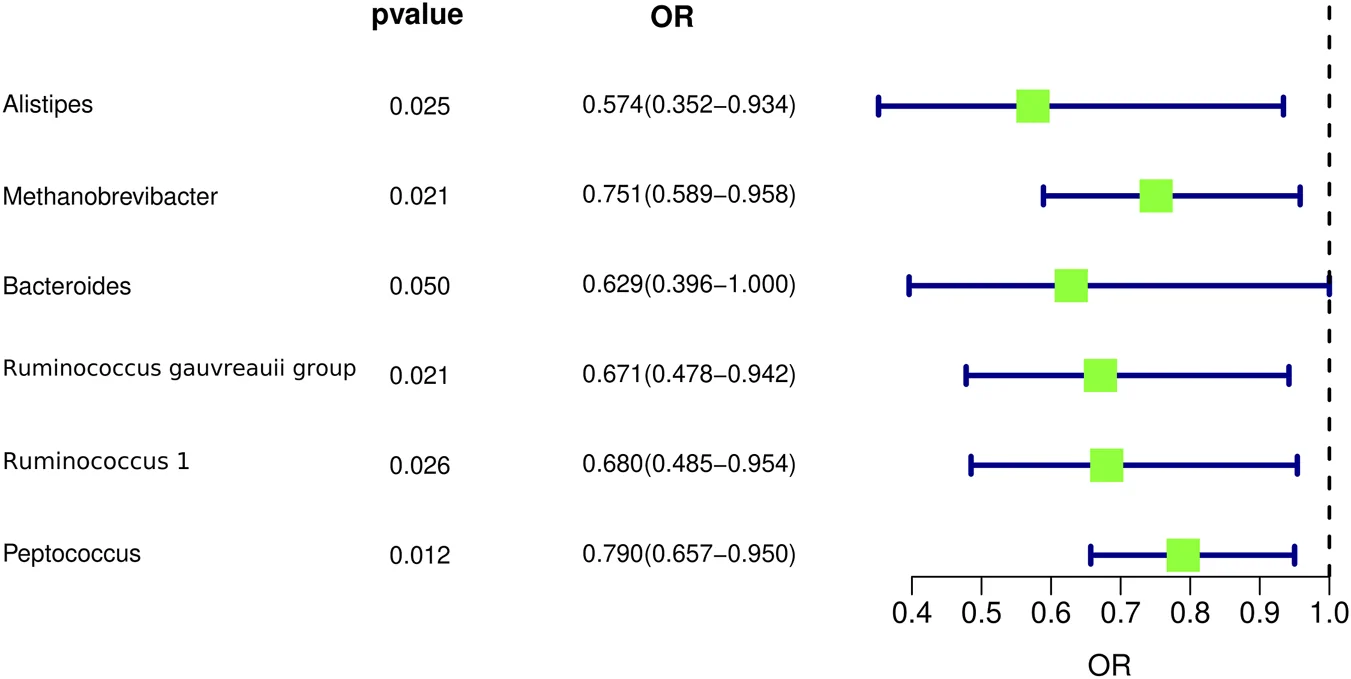
Six nominal inverse IVW associations with NSCLC risk. Points and horizontal lines indicate odds ratios and 95% confidence intervals. P values shown are unadjusted. Table 1 reports BH-FDR q values for these six genera, with all 119 results provided in Supplementary Table S1. None meets q < 0.05. The *Bacteroides* upper confidence limit rounds to 1.000.

### Comparison of MR estimators

Weighted median estimates were inverse for all six genera, but only *Peptococcus* reached nominal P < 0.05 with this estimator (P = 0.0259). MR-Egger estimates were not nominally significant for any of the six genera; the *Bacteroides* MR-Egger point estimate was positive, whereas its IVW estimate was inverse (Figure 3). Simple and weighted mode estimates were also not nominally significant for these genera. Full estimates are provided in Supplementary Table S2.

**FIGURE 3:**
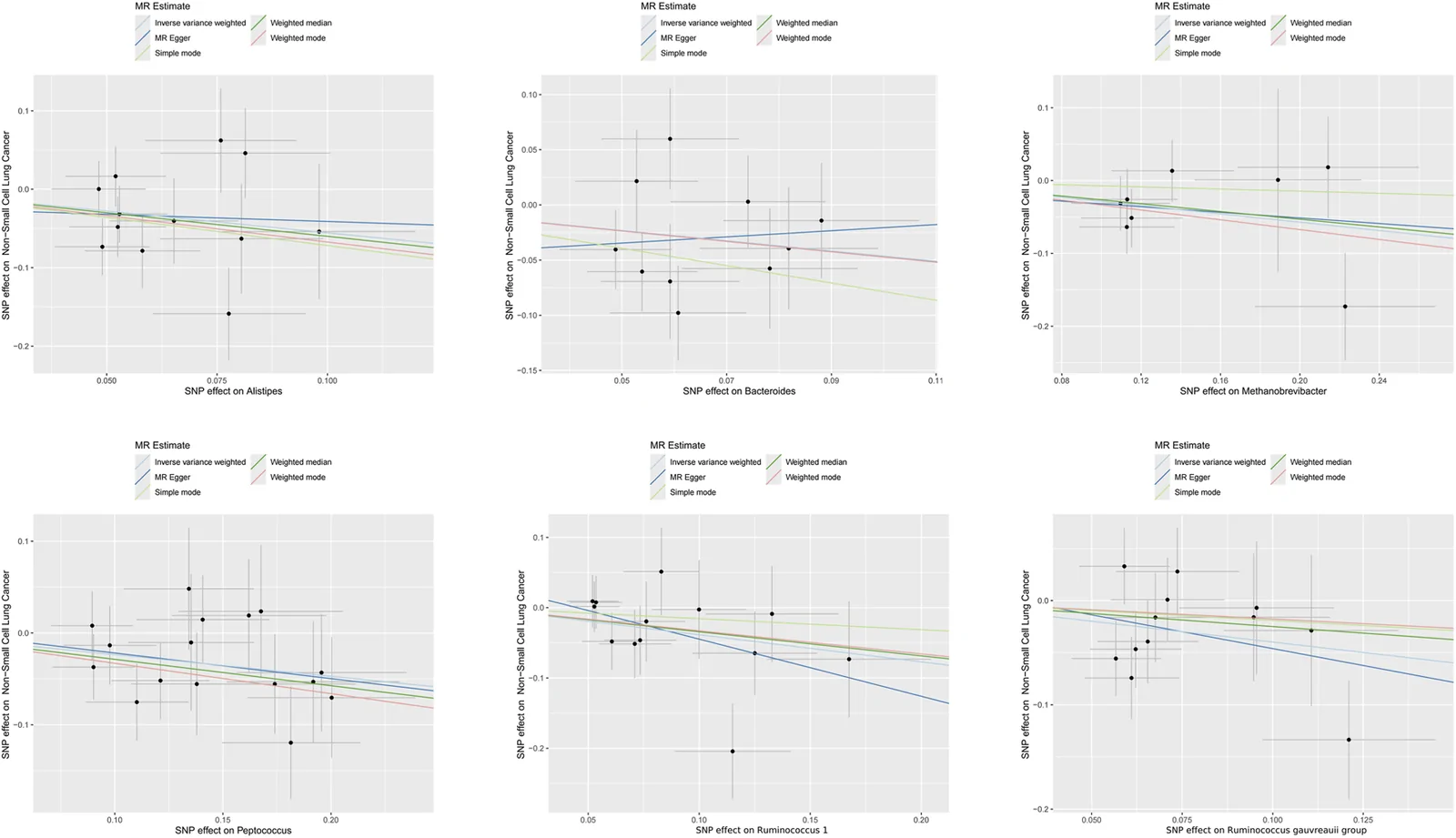
Scatter plots of SNP effects and MR estimates for the six nominally associated genera. Lines represent IVW, MR-Egger, weighted median, simple mode and weighted mode estimates.

### Sensitivity analyses

For the six genera, IVW Cochran’s Q P values ranged from 0.2469 to 0.8964, MR-Egger intercept P values from 0.3582 to 0.8993, and MR-PRESSO global P values from 0.249 to 0.901 (Table 2; Supplementary Table S5). These tests did not detect heterogeneity or directional pleiotropy. Leave-one-out point estimates remained inverse; however, 95% CIs crossed zero after removing some SNPs for Alistipes (4 of 12 exclusions), Methanobrevibacter (2 of 8), *Bacteroides* (7 of 10), Ruminococcus gauvreauii group (3 of 12) and Ruminococcus 1 (1 of 13). *Peptococcus* retained nominal significance across all 16 exclusions (Figure 4; Supplementary Table S6). Funnel plots and single-SNP estimates are shown in Figures 5, 6. Figure 7 outlines a biological framework for further investigation.

**FIGURE 4:**
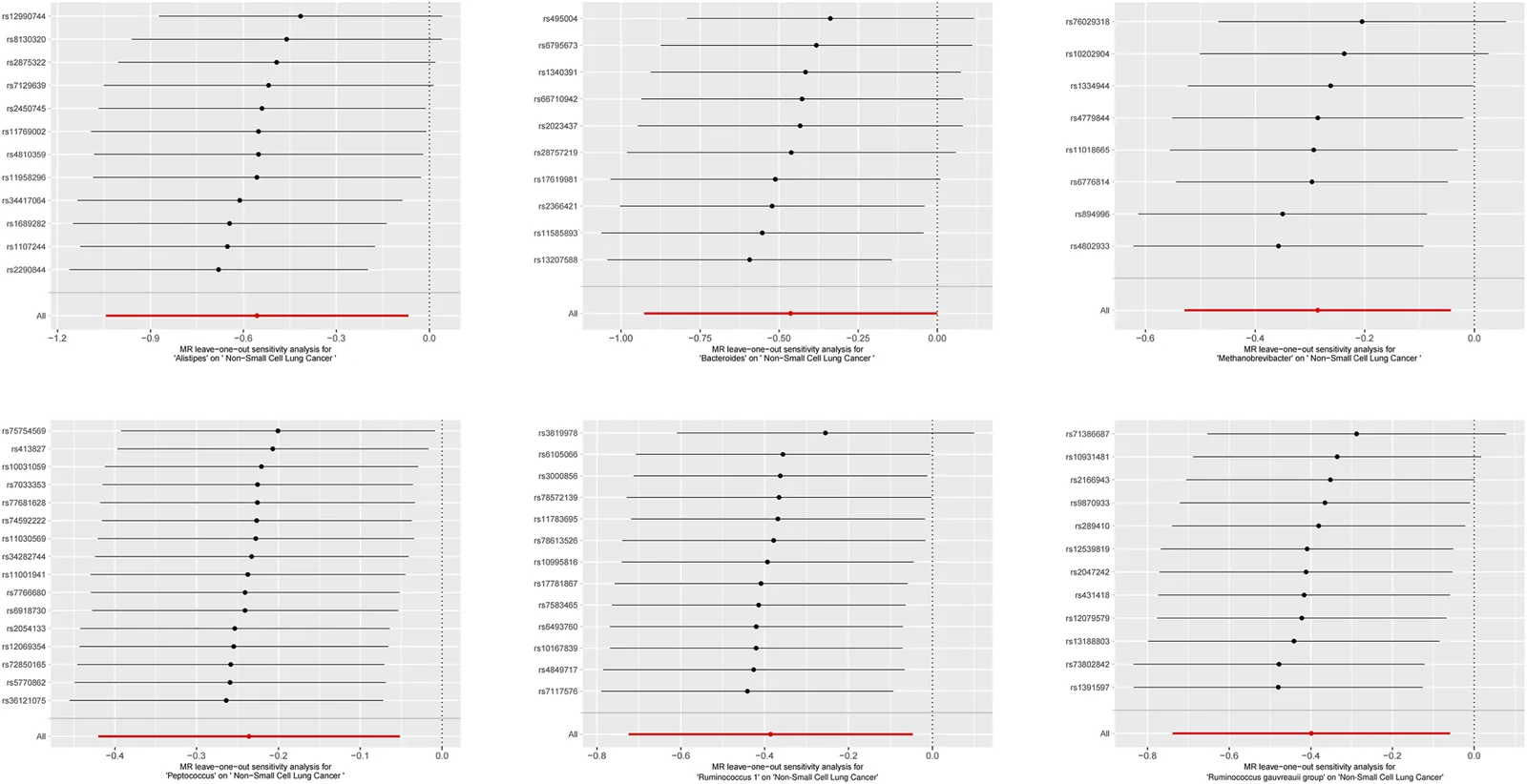
Leave-one-out IVW plots for Alistipes, *Bacteroides*, Methanobrevibacter, *Peptococcus*, Ruminococcus 1 and Ruminococcus gauvreauii group, arranged from left to right across the top and bottom rows. Numerical results are provided in Supplementary Table S6. Each row omits the indicated SNP; the final row uses all instruments. Points and horizontal lines show log odds ratio estimates and 95% confidence intervals. The dashed line indicates the null.

**FIGURE 5:**
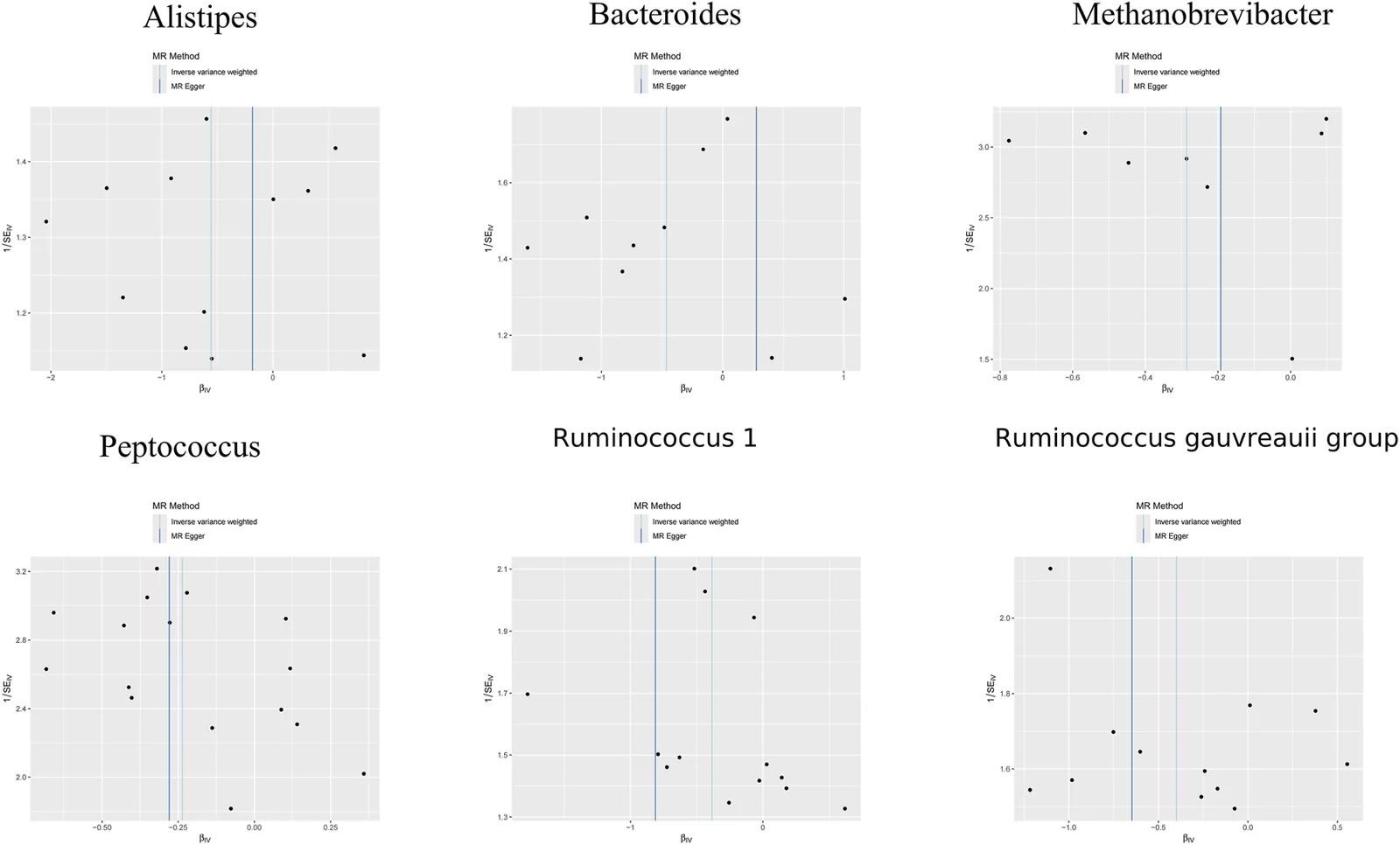
Funnel plots of single-SNP Wald ratios and precision. Vertical lines indicate IVW and MR-Egger estimates.

**FIGURE 6:**
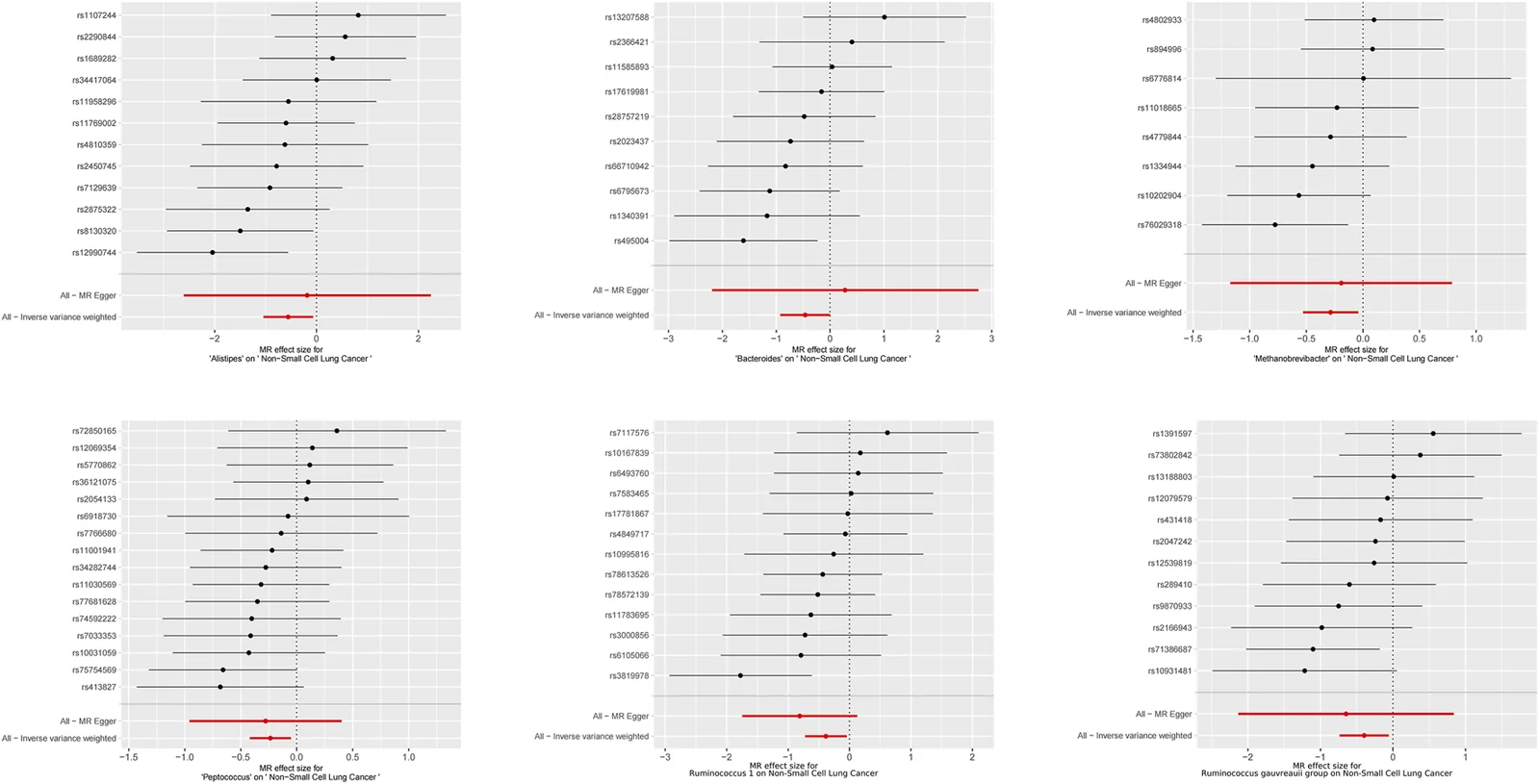
Single-SNP Wald ratio estimates and pooled MR estimates. Horizontal lines indicate 95% confidence intervals on the log odds ratio scale. Individual estimates can differ in direction and precision.

**FIGURE 7:**
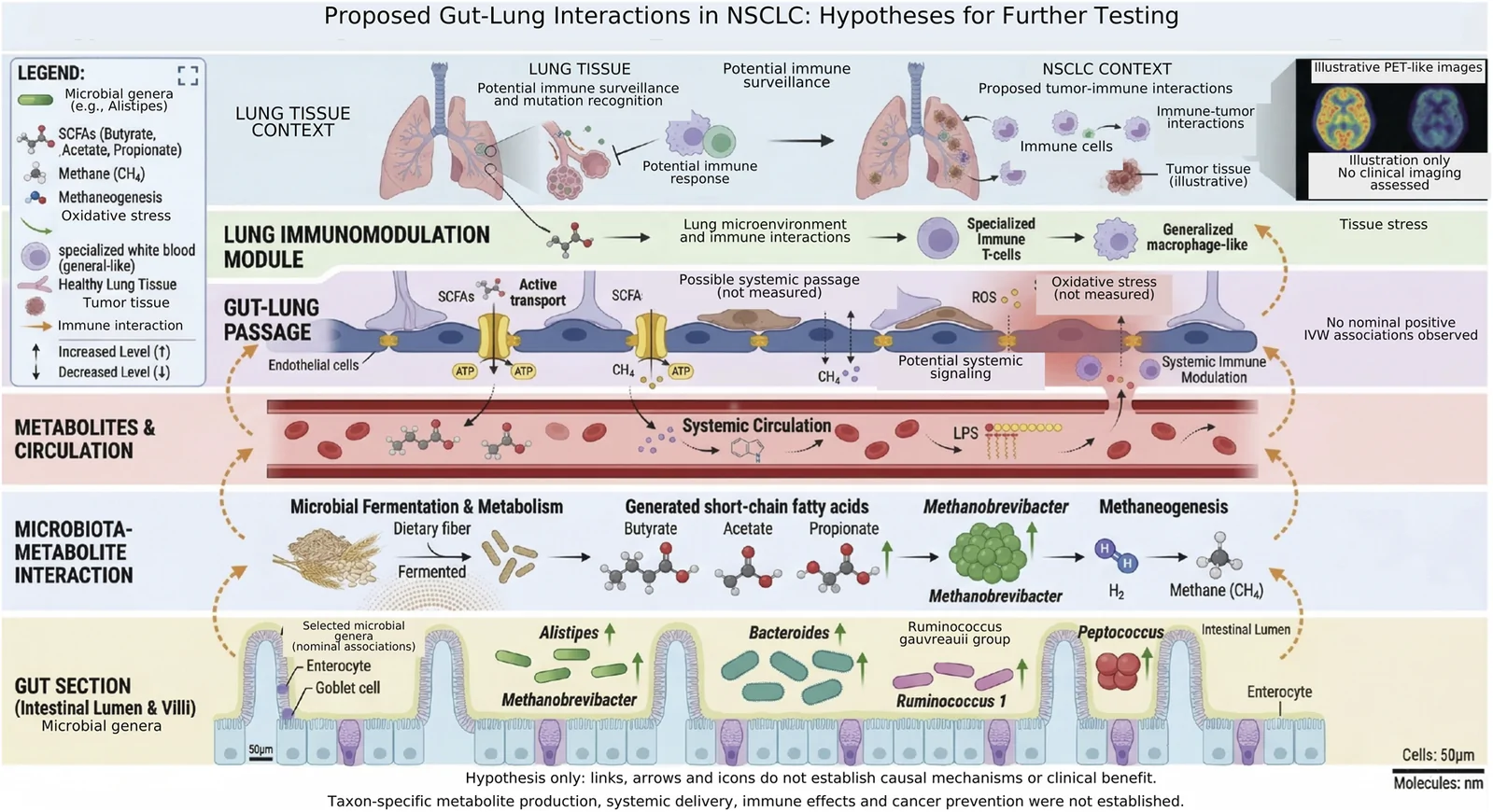
Proposed gut–lung biological framework. The diagram summarizes hypotheses concerning microbial metabolites and lung immune responses for further investigation. The illustrated pathways were not directly tested by the MR analysis or the NaB experiments.

### Exploratory sodium butyrate experiments

NaB was evaluated as a representative microbial metabolite in a separate cell-culture experiment using THP-1-derived macrophages.

In THP-1-derived macrophages, TCM reduced TNF-α, CD86 and iNOS transcript levels and increased Arg-1, TGF-β and CD206 relative to Control. Addition of 1 mM NaB shifted these markers in the opposite direction (Figures 8A–F; n = 4 per group). Tukey-adjusted P values were <0.0001 for both TCM versus Control and TCM plus NaB versus TCM for all six markers. Relative to Control, Arg-1, TGF-β and CD206 remained higher in the TCM plus NaB group (Tukey-adjusted P = 0.0140, 0.00668 and 0.00502, respectively).

**FIGURE 8:**
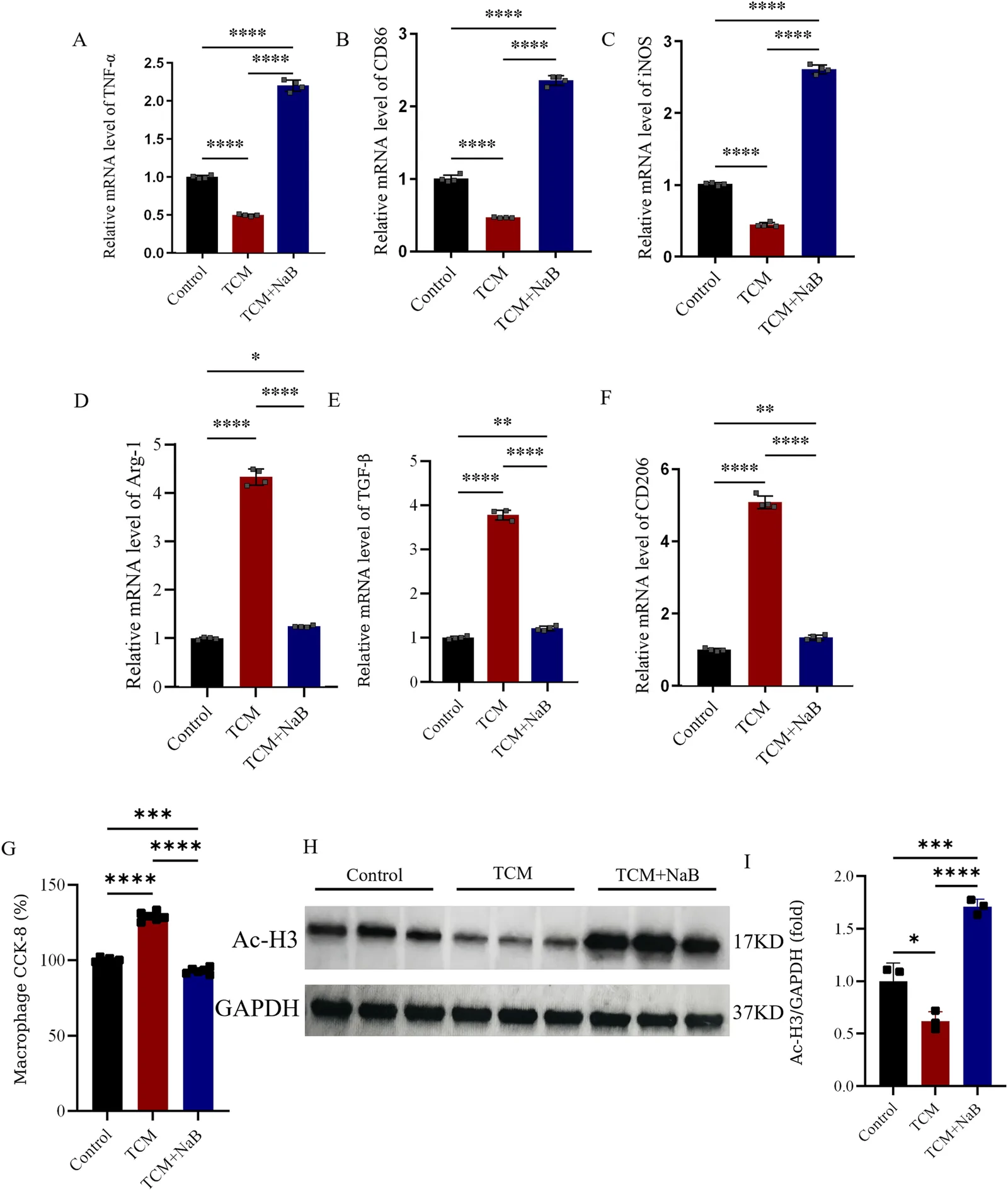
Exploratory NaB experiments in THP-1-derived macrophages. **(A–F)** Relative transcript levels of TNF-α, CD86, iNOS, Arg-1, TGF-β and CD206 measured by RT-qPCR (n = 4 independent biological replicates per group). **(G)** Background-corrected CCK-8 signal as a percentage of the control mean (n = 6 independent biological replicates per group). A549 cells were used to produce TCM; the CCK-8 assay was conducted on macrophages. **(H)** Representative Ac-H3 and GAPDH immunoblots. **(I)** Ac-H3/GAPDH densitometry normalized to the control mean (n = 3 independent biological replicates per group). The groups were Control, 50% TCM and 50% TCM plus 1 mM NaB, with simultaneous treatment for 24 h. Bars and error bars indicate mean ± SD, and points indicate individual biological replicates. qPCR values use sample-matched GAPDH normalization. One-way ANOVA was followed by Tukey’s test for the three pairwise group comparisons within each endpoint, using relative expression, normalized CCK-8 values or normalized Ac-H3/GAPDH values as appropriate. *P < 0.05, **P < 0.01, ***P < 0.001, ****P < 0.0001. Exact values are provided in Supplementary Tables S12–S14.

The CCK-8 assay measured THP-1-derived macrophage responses (Figure 8G; n = 6 per group). Mean relative CCK-8 signals were 100.00% ± 1.51%, 129.10% ± 2.89% and 92.95% ± 1.98% for Control, TCM and TCM plus NaB, respectively. TCM increased the signal relative to Control (Tukey-adjusted P < 0.0001), whereas NaB reduced it relative to TCM (P < 0.0001) and Control (P = 0.000158).

Western blot densitometry in THP-1-derived macrophages showed mean Ac-H3/GAPDH fold changes of 1.00 ± 0.17, 0.62 ± 0.09 and 1.71 ± 0.07 in Control, TCM and TCM plus NaB, respectively (Figures 8H,I; n = 3 per group). TCM reduced the signal relative to Control (Tukey-adjusted P = 0.0190), and NaB increased it relative to TCM (P = 0.0000765) and Control (P = 0.000844).

## Discussion

The MR analysis identified six nominal inverse associations between microbial genus abundance and NSCLC risk. Although none met the multiple-testing threshold, these results provide candidates for independent replication. The direction of the weighted median estimates was consistent with IVW, while statistical support varied across estimators and leave-one-out analyses. This pattern supports treating the associations as suggestive rather than established protective effects.

The distinction between microbial abundance and metabolite function is central to interpretation. MiBioGen genus-level summary statistics do not measure butyrate-production genes or metabolite concentrations (Kurilshikov et al., 2021). Species and strains within a genus can differ in metabolic capacity, and community cross-feeding complicates assignment of a metabolite to one genus. Methanobrevibacter is an archaeal genus. The NaB experiments therefore examine a representative microbial metabolite; they do not establish direct butyrate production by the six genera or mediation of their MR associations.

NaB shifted polarization-associated transcripts in TCM-exposed macrophages and increased Ac-H3/GAPDH, suggesting that microbial metabolites can influence macrophage responses under these culture conditions. The accompanying reduction in CCK-8 signal may reflect changes in cellular metabolism, cell number or viability. A549 cells served as the source of TCM; cancer-cell viability and macrophage-mediated cancer-cell killing were not assessed. Published work has identified HDAC-related effects of butyrate in intestinal macrophages (Chang et al., 2014). Increased Ac-H3 in the present model is consistent with altered histone acetylation, but Ac-H3/GAPDH does not distinguish changes in histone acetylation from changes in total H3 abundance. Direct HDAC activity measurements, total-H3 normalization and receptor perturbation would help distinguish HDAC-dependent effects from GPR41/GPR43 signaling.

Physiological exposure also informs interpretation of the NaB experiments. Gut-derived butyrate is used by intestinal cells and undergoes substantial splanchnic metabolism before reaching systemic tissues. A human stable-isotope study reported approximately 2% systemic availability of colon-delivered butyrate (Boets et al., 2017). Our experiments used a single pharmacological concentration of 1 mM; plasma and tissue concentrations were not measured. Concentration–response studies and direct exposure measurements would clarify whether comparable effects occur at concentrations attainable in the lung.

Clinical studies linking the microbiome to immune checkpoint inhibitor outcomes provide a related context (Routy et al., 2018; Jin et al., 2019; Pinato et al., 2019; Derosa et al., 2018). The present MR analysis addresses NSCLC risk, rather than treatment response, checkpoint inhibitor efficacy, progression-free survival or overall survival. Translation to screening or prevention would require independent validation and evaluation of predictive performance, efficacy and safety. Strain-resolved functional analysis and direct metabolite measurements could help prioritize the associations for further study.

Several design considerations should guide further work. The relaxed instrument threshold may increase susceptibility to weak-instrument bias and winner’s curse despite F statistics above 10. Differences between the multi-ancestry exposure data, Finnish outcome and European LD reference may influence the estimates; participant overlap could not be quantified. Genus-level data limit species- and strain-specific attribution. Reverse MR and Steiger testing were not performed, and the outcome was analyzed as overall NSCLC without histological stratification. Subtype analyses were not added because of concerns about smaller case counts and power. The cellular observations derive from one macrophage cell-line model, one NaB concentration and small biological replicate groups. Functional assays and validation in additional models are needed to assess the persistence, mechanism and biological relevance of these responses.

## Conclusion

This study identifies six suggestive inverse associations between genetically proxied microbial abundance and NSCLC risk, none of which met the multiple-testing threshold. Complementary experiments show that NaB alters polarization-associated transcripts, metabolic viability signals and histone acetylation in TCM-exposed THP-1-derived macrophages. The findings support further investigation of microbial associations and metabolite responses as distinct components of the gut–lung axis.

## Data Availability

The original contributions presented in the study are included in the article/Supplementary Material, further inquiries can be directed to the corresponding author.
